# Pay for performance and contractual choice: the case of general practitioners in England

**DOI:** 10.1186/s13561-017-0142-x

**Published:** 2017-01-31

**Authors:** Eleonora Fichera, Mario Pezzino

**Affiliations:** 1 Manchester Centre for Health Economics, Jean McFarlane Building, University of Manchester, Oxford Road, Manchester, M13 9PL UK; 2 Economics, Arthur Lewis Building, University of Manchester, Oxford Road, Manchester, M13 9PL UK

**Keywords:** P4P, contracts, Primary care, Mobility, I11, I18, J41

## Abstract

The Quality and Outcomes Framework (QOF) is a Pay-for-Performance scheme introduced in England in 2004 to reward primary care providers. This incentive scheme provides financial incentives that reward the overall performance of a practice, not individual effort. Consequently, an important question is how the QOF may affect contractual choices, quality provision and doctor mobility in the primary healthcare labour market. The paper provides a simple theoretical model that shows that the introduction and further strengthening of the scheme may have induced practices to compete for the best doctors and modified their choices in terms of contractual agreements with practitioners. We test the implications of this model using a linkage between Doctors Census data and practices’ characteristics from 2003 to 2007. We use linear multilevel models with random intercept and we account for sample selection. We find that after the introduction of the QOF efficient doctors are more likely to become partners and mobility among doctors has increased. The strengthening of the scheme in 2005 is associated with an increase in the quality of primary care and a reduction in access to the market for new doctors.

## Introduction

The number of GPs in the U.K. has risen by an average of 1.8% each year between 2004 and 2014, but there are still problems in recruiting and retaining GPs into the healthcare market (The Health Foundation [16]). In the U.K. there is also a relative long tradition in the use of Pay-for-Performance (P4P) schemes, but little is known about the sorting and retention effects they might produce (Lazear [9]).

The aim of this paper is to investigate the sorting and retention effects of the only P4P scheme for General Practitioners (GPs) in the U.K., the Quality and Outcomes Framework (QOF). GPs are paid under a mix of capitation and financial incentives including the QOF. The QOF awards an amount of money to practices that achieve certain levels of performance. Such pay is linked to the performance of the practice and may then be internally distributed over to GPs. We examine the effects that the introduction of the QOF in 2004 and its subsequent strengthening in 2005 had on the contractual arrangements, the provision of quality and doctor mobility in the primary healthcare market.

We motivate our empirical analysis with a simple theoretical model with two practices combining three aspects of the primary healthcare sector that together, to our knowledge, have not yet been considered by the literature. These are: i) the altruistic^1^ nature of the GP’s job; ii) different contractual arrangements (e.g. salaried positions or partnerships) for GPs (National Audit Office [11]); and iii) the multidimensional nature of quality experienced by patients as a mix of the effort of GPs, front desk and nursing staff. In our model each practice serves a representative patient and requires the effort of one doctor to provide healthcare. Doctors may be of two types: efficient and inefficient. If they are efficient, then they are able to provide medical effort at a lower cost than the other type of doctors, the inefficient ones. The model predicts that the behaviour of a salaried doctor will not be affected by the introduction of the P4P scheme, since her remuneration is not quality-dependent. However, the introduction of the QOF may induce practices to compete (offering partnerships) for the best doctors and increase mobility.

We test the empirical implications of this model using a unique linkage between GP Census data from 2003 to 2007 with practices’ characteristics in England. We then link these data with information on practices’ performance on the QOF. Whilst we find that the introduction of the QOF in 2004 decreased the probability of becoming a partner by one percentage point, more efficient doctors are more likely to be partners. Mobility of doctors increased by two percentage points after the QOF was introduced. But more efficient doctors are less likely to move. And doctors in better practices are also less likely to move. Overall, these results might suggest some evidence of a sorting effect of the QOF with more efficient doctors becoming partners and retention of doctors in higher quality practices. Finally, we find that the strengthening of the scheme in 2005 has produced on average an increase in primary care quality of practices. Over the period between 2004 and 2007 more efficient doctors improve the quality of the practice and if they are also partners they put more effort. However, we observe a *decrease* in GPs’ effort connected to the strengthening of the scheme. We interpret this result with the existence of reputational effects as described by the literature on pro-social behaviour and intrinsic motivation.

## Background

### Literature

Incentive pay is a way to partially overcome information asymmetries in the labour market. The rationale behind P4P is to tie workers’ compensation to their output aligning their individual objectives to those of the firm (see for example, Lazear [9]; Prendergast [13]; Ross [15]). If such schemes are introduced in multi-level organisations, there might be two potentially counteracting effects at play. On the one hand, individual agents might free-ride on the team thus weakening the incentive effect of P4P. On the other hand, the sorting effect might cause more able workers to select and stay in performance pay jobs and less able workers to leave these jobs (see for example, Dohmen and Falk [4]; Gielen et al. [6]; Lazear [10]; 2000). According to these studies, in equilibrium workers relocate themselves on the basis of their ability, thus increasing productivity and wages in firms that use P4P schemes.

In this paper we focus on the QOF, an incentive scheme that applied to practices, which has increased doctors’ income by 25 percent (NHS Review Body [12]). We examine its effects on doctors’ behaviour (e.g. mobility and effort provision) and on practices’ behaviour (e.g. contractual arrangements). This is an important issue for the incentive and sorting effects in the GPs’ labour market and for the resulting potential inequalities in the provision of healthcare to patients.

### The Quality and Outcomes Framework

In the U.K. most healthcare is delivered by the National Health Service (NHS), which is a comprehensive freely available service funded out of general taxation. All citizens are registered with a GP who delivers primary care and acts as gatekeeper for elective secondary care. Primary care practices are typically a group of one to six doctors responsible for a pool of patients. They are independent contractors retaining the surpluses they make as private income.

In early 2000s the terms of two new contracts for GPs remuneration were negotiated (see the National Audit Office [11]). The first contract is the nationally negotiated General Medical Services (GMS), with a mix of capitation, lump sum allowances and P4P schemes, such as the QOF. The capitation payment is based on the number of patients treated by a practice, adjusted for the characteristics of both patients and area where the practice operates. The second contract is the Primary Medical Services (PMS), negotiated between the practice and the local primary care organisation. Under the PMS contract, the practice receives a lump sum payment for the same services provided in the GMS contract (Gravelle et al., [8]). The first three years of this new contract cost about £1.76 billion (National Audit Office [11]).

The new GMS contract was officially introduced on 1^*st*^ April 2004 with measurement of achievements on 1^*st*^ April 2005 for the previous 12 months (Roland [14]). In the first two years of the QOF, practices were rewarded on the basis of performance points reported on 146 indicators. The points correspond to an amount of money in pounds sterling (£) claimed annually. Points were awarded on the basis of reported coverage rates above a lower threshold of 40% and an upper threshold that varied depending on the particular indicator (see Doran et al., [5]). The average price per point in 2004 was £76, increasing to £125 in 2005. Thresholds of payments for some indicators increased between 2005 and 2006 so that it was more difficult for a practice to be rewarded the same amount in 2006 as in 2005 if their performance did not change.

## Methods

### The theoretical model

Let us consider a market where patients are treated by two practices, say practice 1 and 2, located at a positive distance *t*>0 from each other. Practices need only one doctor to serve their population. Let us suppose that there are two types of doctors: efficient (*v*) and inefficient (*nv*). The mass of doctors of type *v* is equal to *π*∈[0,1], and consequently the mass of doctors of type *nv* is equal to (1−*π*).

Suppose that the practices know the type of two doctors, each located next to a practice. These doctors’ type may be known because they were previously employed.

Practices can however access the labour market and offer a salary to new doctors (whose type will be ex-ante unknown) who receive salary *w*_*i*_ from practice *i*, *i*=1,2. The utility of the salaried doctor *z*={*v*,*n* *v*}, who works for practice *i*, is (superscript *N* indicates that we are considering the case of new salaried doctors):

1 $$ U_{zi}^{N}=w_{i}+Q_{zi}-\gamma_{z}\frac{e_{zi}^{2}}{2}+b_{i}   $$

where *Q*_*zi*_ is the total level of quality of care of practice *i*; $\gamma _{z}\frac {e_{zi}^{2}}{2}$ is the doctor’s cost of effort^2^, with 1=*γ*_*nv*_≥*γ*_*v*_>0 representing the degree of inefficiency of doctor *z*; *e*_*i*_ is the level of effort invested by the doctor employed by practice *i* and *b*_*i*_≥0 is the benefit the doctor receives from accessing the amenities in the neighbourhood of practice *i*. Let us assume that the outside option of a new salaried doctor is equal to zero.

Notice the utility in (1) increases with the quality of care *Q*_*zi*_; this captures the assumption that doctors are assumed to be altruistic and care about quality. The quality perceived by patients is the sum of quality improvements given by doctor’s effort and practice’s investment in facilities and nursing staff:

2 $$ Q_{zi}=q_{i}+e_{zi}  $$

where *q*_*i*_ is the quality investment of the practice (e.g. nursing staff, front desk, IT).

Practices are run by doctors who have taken a completely profit-oriented managerial position in the practice.

If a practice employs a new doctor its (expected) profit function is given by:

3 $$ \Pi_{i}^{N}=m+p\left[ \pi Q_{vi}+(1-\pi)Q_{nvi}\right] -w_{i}-\frac {q_{i}^{2}}{2}   $$

where *m* is a capitation payment, *p*≥0 is the quality-related payment based on a P4P scheme, $\frac {q_{i}^{2}}{2}$ is the cost function of the practice^3^.

The timing unravels as follows.

In stage 1, practices (i.e. managers) choose the contract to offer to the doctor; specifically they may offer a partnership to one of the doctors whose type is known or a salaried contract to a new doctor.

In stage 2, practices choose investment in quality *q*_*i*_ to maximise profits. Doctors who accepted a contract choose the level of effort *e*_*zi*_ to maximise utility.

In stage 3, primary health care is provided.

#### Salaried contract

Let us look first at the provision of care when a practice hires a new doctor.

The effort chosen by the salaried doctor in stage two is simply produced by the satisfaction of first order conditions to maximise (1).

4 $$ \frac{dU_{i}^{N}}{de_{zi}}=0\Rightarrow e_{zi}^{N}=\frac{1}{\gamma_{z}}   $$

Notice that *p* has no effect on the effort choice of the salaried doctor. This is not surprising, since she receives a remuneration that does not depend on the quality of care.

Similarly the maximisation of profits defines the investment in quality of the practices at stage two.

5 $$ \frac{d\Pi_{i}^{N}}{dq_{i}}=0\Rightarrow q_{i}^{N}=p   $$

Since the outside option of a new doctor is equal to zero and the resulting utility is strictly positive, practices will set *w*_*i*_=0.^4^ Notice that a positive *p* is necessary to induce the practice to invest in quality. In equilibrium:

6 $$ \Pi_{i}^{N}=\Pi^{N}=m+\frac{p}{2}^{2}+\frac{p(\gamma_{v}+\pi-\gamma_{v}\pi)}{\gamma_{v}}   $$

Notice also that, when the practices choose to hire a new doctor, profits increase in *m*,*p*, and *π*, and decrease in *γ*_*v*_. In addition, if *p*=0 (i.e. no P4P scheme is in place), then $\Pi _{i}^{N}=m$.

#### Partnership contracts and competition over doctors

Subsection Salaried contract considered the case in which practices decided to hire a new doctor in stage one. However next to each practice lives a doctor whose type is known and who could be offered a partnership. A practice may be also willing to pay a relocation package equal to *t* and offer a partnership to the doctor located next to the other practice. Similar to the analysis in section Salaried contract, let us study first the provision of care of a practice who employs a partner of type *z*.

Assuming that a partner will be entitled, in addition to a salary, to equally share profits with the management,^5^ the utility of a partner and the remaining profits for the practice are equal to:

$$\begin{array}{*{20}l} {}U_{zi}^{P} & =\frac{1}{2}\left[ m+pQ_{zi}^{P}-w_{i}^{P}-\frac{q_{i}^{2}} {2}\right] \!+w_{i}^{P}+Q_{zi}^{P}-\gamma_{z}\frac{e_{zi}^{2}}{2}+b_{i}\\ {}\Pi_{zi}^{M} & =\frac{1}{2}\left[ m+pQ_{zi}^{P}-w_{i}^{Pi}-\frac{q_{i}^{2} }{2}\right] \end{array} $$

Superscripts *P* and *M* indicate variables respectively related to a doctor working as a partner and a manager in a partnership.

The simultaneous choice of medical effort and practice quality produces:

7 $$ \left\{ \begin{array} [c]{c} \frac{d\Pi_{zi}^{M}}{dq_{i}}=0\\ \frac{dU_{zi}^{P}}{de_{zi}}=0 \end{array} \right. \Rightarrow\left. \begin{array} [c]{c} q_{zi}^{M}=p\\ e_{zi}^{P}=\frac{2+p}{2\gamma_{z}} \end{array} \right.   $$

Comparing (7) with (4) it is clear that $e_{zi}^{P}\geq e_{zi}^{N}$ (this is the effect of the incentive of the P4P scheme). Not surprisingly, the practice investment is not directly affected by the partnership and $q_{zi}^{M}=q_{i}^{N}$. In addition, without the P4P scheme, i.e. *p*=0, practice quality would be $q_{zi}^{N}=q_{zi}^{M}=0$.

The study of the expressions in (4), (5) and (7) produces a testable implication: *an increase in the QOF price per-quality-point (i.e. higher* *p* *) may increase medical effort and practice quality*.

The utility of the partner in equilibrium is given by

$$U_{zi}^{P}=b_{i}+\frac{(p+2)^{2}}{8\gamma_{z}}+\frac{(2m+p^{2}+2w_{i}^{P} +4p)}{4} $$

$U_{zi}^{P}>0$. Let us suppose again that the outside option of the doctor is sufficiently low to allow practices to offer $w_{i}^{P}=0$. Remaining equilibrium profits for the manager are given by

$$\Pi_{zi}^{M}=\frac{\gamma_{z}(2m+p^{2})+p(p+2)}{4\gamma_{z}} $$

An increase in *p* (P4P incentive) has a positive effect on quality (both $Q_{zi}^{P}$ and $e_{zi}^{P}$), partner’s utility and practice’s profits. Notice that if *p*=0 (i.e. no P4P scheme is in place) then $\Pi _{zi}^{M} =\frac {m}{2}<\Pi ^{N}=m$. In other words, without a P4P scheme, it is not desirable for a profit-oriented practice to offer a partnership. This implies that with the introduction of P4P incentives practices become more concerned about the ability of the doctors. Consequently, they will consider offering partnerships and eventually compete to attract the best doctors.

This provides another testable implication: *the introduction of the QOF (* *p*>0*compared to* *p*=0*) may have created mobility among doctors.*

In stage one a practice needs to choose whether to offer a partnership to (either) of the two old doctors or access the labor market.

Three scenarios may exist: (i) both old doctors are of type *v*, (ii) both old doctors are of type *nv*, (iii) the old doctor in practice 1 is of type *v*, while the old doctor in practice 2 is of type *nv*.^6^ Due to the existence of relocation costs *t*, in cases (i) and (ii) the practices will not find profitable to offer a partnership to the doctors located in another region. There is not competition for doctors is case (i) and (ii) and, therefore, we discard these scenarios. Relocation will be feasible instead in case (iii) and this will be the focus of our analysis.

In any case, in stage one a practice will consider to offer a partnership to the local doctor if $\Pi _{zi}^{M}>\max \left \{ 0,\Pi ^{N}\right \} $. The necessary condition is satisfied in the subset of the parameters *S*, where:

$${{ \begin{aligned} {} S & \equiv S_{1}\cup S_{2}\\ {}& \text{and}\\ {}S_{1} & =\left[ \left. \left(m,p,\!\gamma_{z}\right) \right\vert\! 0\!\leq\! m\!<\!\bar{m},p\!>\!\bar{p},\!0\!<\!\gamma_{z}\!<\!1,\!\frac{1\,-\,2\gamma_{z}}{2\!\left(1\,-\,\gamma_{z}\right)}\!<\!\pi\!<\!1\!\right] \\ {}S_{2} & =\left[ \left. \!\left(m,p,\!\gamma_{z}\right) \right\vert\! 0\!\leq\! m\!<\!\bar{m},p\!>\!0,\!0\!<\!\gamma_{z}\!<\!1,0\!<\!\pi\!<\!\frac{1\,-\,2\gamma_{z}}{2\left(1\,-\,\gamma_{z}\right) }\!\right] \\ {}\bar{m} & =\frac{p(p-\gamma_{z}p+2(1+2\gamma_{z}(-1+\pi)-2\pi))}{2\gamma_{z}}\\ {}&\text{and }\bar{p}=\frac{2(2\pi-1-2\gamma_{z}(1-\pi))}{1-\gamma_{z}} \end{aligned}}} $$

It follows that a practice will offer a partnership only if the probability to hire an efficient new doctor is low or if the P4P incentive is sufficiently strong to enhance the performance for the doctor and, consequently, profits. Notice that *γ*_*nv*_=1 does not belong to sets *S*_1_ nor *S*_2_. This means that $\Pi _{nvi}^{M}<\Pi ^{N}$. In other words, no practice will offer a partnership to an inefficient doctor.

This provides another testable implication: *efficient doctors are more likely to be offered a partnership*.

Focusing our attention on parameters in *S*, simple and intuitive comparative statics analysis on $\left (\Pi _{vi}^{M}-\Pi ^{N}\right) $ shows that an increase in *π* (the probability to hire an efficient new doctor), in *γ*_*v*_ (i.e. less efficient to provide effort for type *v* doctors) or in the capitation payment *m* reduce the profitability of offering a partnership and may increase turn-over. An increase in *p*, instead, induces a practice to offer a partnership to the local efficient doctor. This last result implies that *an increase in* *p* *reduces access to the job market to new (salaried) doctors*, another testable implication.

Let us focus our attention to the case in which an asymmetric allocation of doctors may induce practices to compete for the best doctors. Let us assume for example that an efficient doctor is located next to practice 1 and an inefficient one is located next to practice 2.

Let us restrict our attention to the following subset of parameters:

$$\hat{S}\equiv S\cap\left[ \left. t\right\vert 0<t<\Pi_{vi}^{M}-\Pi_{nvi} ^{M}\right] $$

Set $\hat {S}$ ensures that relocation costs are sufficiently small to make relocation of an efficient doctor feasible.

Practice 1 may face competition from practice 2 over the services of the efficient doctor and therefore may have to offer a higher salary in order to outbid the rival. Define $\tilde {\Pi }_{v1}^{M}$ the profits earned by practice 1 hiring the efficient doctor when practice 2 is also offering a partnership to the same doctor. Practice 1 will offer a partnership to doctor *v* if:

$$\tilde{\Pi}_{v1}^{M}\geq\max\left\{ 0,\Pi^{N}\right\} $$

where

$$\begin{array}{*{20}l} \tilde{\Pi}_{v1}^{M} & =\max\Pi_{v1}^{M}\\ \text{s.t.} U_{v1}^{P}\left(w_{1}\right) & \geq U_{v2}^{P}\left(w_{2}\right) \end{array} $$

Constraint $U_{v1}^{P}\left (w_{1}\right) \geq U_{v2}^{P}\left (w_{2}\right) $ requires that the efficient doctor will not be worse off accepting a partnership by practice 1 rather than practice 2. Using expressions (7), the condition simplifies to

8 $$ w_{1}\geq w_{2}-2(b_{1}-b_{2})  $$

The highest salary that practice 2 will be willing to offer to the vocational doctor is

9 $$ \bar{w}_{2}^{{}}:\max\Pi_{v2}^{M}-t=\max\Pi_{{}}^{N}  $$

It follows that $\bar {w}_{2}=\frac {p^{2}-\gamma _{v}(2m+p^{2}+4t)+2p(1+2\gamma _{v}(-1+\pi)-2\pi)}{2\gamma _{v}}\geq 0$ for parameters in $\hat {S}$.

The salary that practice 1 has to guarantee to the local efficient doctor to beat competition from practice 2 is

$${{ \begin{aligned} {} \bar{w}_{1} & \,=\,\bar{w}_{2}-2(b_{1}-b_{2})\\ {}& \,=\,\frac{p^{2}\,-\,\gamma_{v}(4b_{1}\,-\,4b_{2}\,+\,2m\,+\,p^{2}\,+\,4t)\,+\,2p(1\,+\,2\gamma_{v} (-1\,+\,\pi)\,-\,2\pi)}{2\gamma_{v}} \end{aligned}}} $$

Simple comparative statics analysis shows that $\frac {d\bar {w}_{1}}{dp}>0$ (an increase in *p* increases the performance of the partner and, therefore, the willingness to pay of practice 2) and $\frac {d\bar {w}_{1}}{db_{2}}>0$ (if the neighbourhood of practice 2 provides attractive amenities, practice 1 needs to reward the efficient doctor more to convince her to stay); also $\frac {d\bar {w}_{1}}{dm}<0$, $\frac {d\bar {w}_{1}}{d\gamma _{v}}<0$, $\frac {d\bar {w}_{1}}{d\pi }<0$ (increases in *m*, *γ*_*v*_ and *π* make less desirable for practice 2 the option of hiring a new doctor and, consequently, practice 1 can reduce the salary offered to the efficient doctor^7^); $\frac {d\bar {w}_{1}}{dt}<0 $, $\frac {d\bar {w}_{1}}{db_{1}}<0$ (an increase in *t* or *b*_1_ makes more difficult for practice 2 to attract the efficient doctor).

The profits obtained by practice 1 when offering the partnership to the local efficient doctor are:

10 $$ \tilde{\Pi}_{v1}^{M}=b_{1}-b_{2}+m+t+\frac{p}{2}\frac{p+2(\gamma_{v} +\pi-\gamma_{v}\pi)}{\gamma_{v}}  $$

Finally

11 $$ \tilde{\Pi}_{v1}^{M}-\Pi^{N}=b_{1}-b_{2}+t  $$

Mobility (in our example the relocation of the efficient doctor from practice 1 to practice 2) ultimately depends on the relocation costs and the characteristics of the areas where the two practices operate. Indeed mobility will increase if the relocation costs or the workplace quality differential (*b*_1_−*b*_2_) decrease. The P4P scheme does not directly affect the willingness to pay of practice 1 to retain the efficient doctor. Since the scheme offers the same incentives to both practices, an increase in *p* equally induces both practices to compete for the efficient doctor. The scheme, however, may have an effect on the welfare of the efficient doctor. In fact, the efficient doctor may be better off because of the competition from practice 2. The increase in utility (compared to a partnership in practice 1 without competition) depends on ultimately in $\bar {w}_{1}$.

### Empirical strategy

The above-described theoretical model motivates our empirical strategy by providing the following testable implications:

1. Efficient doctors are more likely to be offered a partnership.
2. The introduction of the QOF (*p*>0 compared to *p*=0) may have created mobility among doctors.
3. An increase in the QOF price per-quality-point (i.e. higher *p*) may have (i) increased medical effort and practice quality, (ii) decreased access to the job market to new (salaried) doctors to replace inefficient colleagues.

As a result, the empirical strategy focuses on how to model efficiency, partnership, mobility and access of new doctors to the healthcare market. Efficiency can be modelled as a function of an observed component, e.g. experience, and an unobserved one, e.g. ability. Ideally, we would need a single data source with a large longitudinal sample where we have sufficient doctors’ mobility and we measure wage as proxy for efficiency. Since such source of data is not available, we have to use age as proxy for efficiency in the General Medical Services (GMS) Statistics Databases. In reality there are complex interactions between experience and efficiency so it is not an unreasonable assumption. We can test whether age is positively correlated with practices’ quality on a sample of single-handed practices in order to attribute efficiency to a single doctor rather than to the average doctor in a practice. We estimate three separate pooled linear regression models. These models can be formally described as follows:

12 $$ y_{ijt}=a+b{x}_{ij(t-1)}+d{w}_{ij(t-1)}+g{z}_{j(t-1)}+\epsilon_{ijt}   $$

where *y* indicates the proportion of total QOF points practice *j* achieves. The subscript *i*=1,…,*N* indicates each of the N doctors, *j*=1,…,*N* indicates each practice and *t*=2004,2005. Demographic characteristics of doctor *i*, including age, are denoted by *x*_*ij*(*t*−1)_, *w*_*ij*(*t*−1)_ is doctor’s *i* income in practice *j* and *z*_*j*(*t*−1)_ are practice *j* characteristics. *ε*_*ijt*_ indicates the residual of equation (12). We then test the implications of the theoretical model using pooled linear probability models and random intercept multilevel models^8^, accounting for sample selection bias in the mobility equation.

The first and second testable implications are modelled as the probability of being a partner or to move to another practice:

13 $$ y_{ijt}=a_{i}+b{x}_{ij(t-1)}+g{z}_{j(t-1)}+\epsilon_{ijt}   $$

where as in Eq. 12 *i* and *j* indicate the doctor and practice, respectively; *t*=2003,…,2007, where *y*_*ijt*_=1 indicates whether the doctor is a partner or has moved to another practice. We argue that *a*_*i*_ captures the unobserved component of efficiency, the ability of doctor *i*. The time-variant error term is denoted as *ε*_*ijt*_ which also includes practice effects *d*_*j*_.

To test the second implication of the theory, we model mobility as a two stages Heckman model. The first stage regression is essentially the same as Eq. 13 with *y*_*ijt*_ representing the realised value of doctors’ propensity to stay in the health care job market. The exclusion restriction of the first stage regression is the number of years to statutory retirement depending on the time of the survey. Statutory retirement does not vary by practice and should not affect the probability of changing practice ^9^. From each year’s probit model we calculate the Inverse Mills Ratio (IMR) as the ratio of the probability density function to the cumulative distribution function. We then “stack” all IMRs and include the time-variant IMR into the second stage random intercept multilevel model of mobility. Conditional on staying in the market, doctors might want to stay in the same practice or to move practices which is formalised with the inclusion of the time-varying IMR as additional covariate. In these specifications we include 2003 as a pre-QOF year because we want to test the effect of the introduction of a new performance scheme on the contractual choice and mobility of doctors.

Finally, we test the third implication of the theoretical model by examining whether the price increase induced by the QOF is associated with an increase of medical effort (measured by the workload of a doctor or full-time equivalence (FTE)), practice quality (measured by the proportion of total QOF points) and a decrease in access of new doctors in the market. The first two models can be formalised as follows:

14 $$ y_{ijt}=\alpha_{i}+\beta{x}_{ij(t-1)}+\gamma{z}_{j(t-1)}+\epsilon_{ijt}   $$

where *y*_*ijt*_ indicates doctors’ effort measured by their FTE and the proportion of total QOF points practice *j* achieves. The other covariates are the same as those described above, but *t*=2004,…,2007. The third model is formally the same as the one in Equation 13 with *y*_*ijt*_ indicating whether a new doctor entered the market.

#### Robustness checks

One potential threat to our empirical strategy is the identification of the effect of the QOF on doctors’ mobility from a before and after comparison as the P4P scheme applied to all practices. We can indirectly test the robustness of the results of the 2005 price increase using a similar price change that occurred in 2006. We use the exogenous price shock determined by the increase in threshold levels in 2006, constructed as a potential income loss for each practice, and test its effect on doctors’ effort, access of new doctors in the market and mobility. If the before/after comparison was not biased by other policies then we would expect this price decrease to generate similar results as before (but with opposite sign). We highlight a caveat. As this price decrease only applied to some indicators of cardiovascular diseases (CVD), there could be effort diversion to similar tasks for which payments remained unchanged. As an additional robustness check we consider the full set of CVD indicators for which effort diversion might have occurred. More formally, we estimate:

15 $$ \Delta{y}_{ij}=b{\Delta{x}}_{ij}+\theta{\Delta{S}}_{ij}+g{\Delta{z}} _{j}+\epsilon_{ijt}   $$

where the unobserved component *α*_*i*_ is differenced out and *Δ* *y*_*ij*_ indicates the change in FTE, access to the market of new doctors and mobility. We estimate the change in mobility separately for the top and bottom performers, measured as practice performance in 2004. *Δ* *x*_*ij*_ includes the same characteristics as in Eq. (14), but age. *Δ* *S*_*ij*_ is the exogenous price shock between 2005 and 2006.

Finally, models of partnership, mobility and access to the healthcare job market could be estimated as non-linear models because the outcome variables are binary. The latent propensity to be a partner, to move to another practice or to access the healthcare job market can be modelled as:

16 $$ y_{ijt}^{\ast}=a_{i}+b{x}_{ij(t-1)}+g{z}_{j(t-1)}+\epsilon_{ijt}   $$

where, as in Eq. 12, *i* and *j* indicate the doctor and practice, respectively; *t*=2003,…,2007, and $y_{ijt}=1.(y_{ijt}^{\ast }>0)$.

## Data and summary statistics

### Data sources

Our analysis is based on two data sources: i) the General Practitioners’ Worklife Survey (GP WLS); and ii) the General Medical Services (GMS) Statistics Databases to which we link geodata and measures of practices’ quality. We use multiple data sources to combine their relative strengths because the GP WLS records doctors’ income, but as a small longitudinal sample it does not contain a large sample of doctors who move. The GMS database instead contains information on all doctors in England, but does not record their income.

We use the 2004 and 2005 longitudinal samples of about 2000 doctors from the GP WLS, a postal survey of GPs’ working lives undertaken by the National Primary Care Research and Development Centre. The questionnaire contains information on personal characteristics of the GPs, their job satisfaction and some characteristics of the practices.

The main data source are the GMS Statistics Databases from 2003 to 2007. They are annual Censuses of GPs in post at each September each year. They contain information on practice codes, Primary Care Trusts (PCTs) codes, GPs age, gender and country of qualification; GP type (i.e. salaried or partner), full-time equivalence (FTE) (including hours if available). They also include information on practice contract, whether it is a dispensing practice, its list size by age-group and gender.

Any doctor who treats patients is required by the law to register with the General Medical Council (GMC) to obtain a licence to practice. Once registered, doctors receive a GMC reference number that uniquely identifies them. This number has been used to trace doctors in the yearly Censuses so that our final dataset contains a description of characteristics and movements of doctors from 2003 to 2007.

Practice postcodes have been used to calculate distances and to link local areas characteristics such as the Local Income Scheme Index (LISI) to this data. LISI is a direct measure of practice list deprivation derived from prescription data.

Finally, we link practice codes to the Health and Social Care Information centre database containing numerators, denominators and number of points that each practice has obtained on all QOF indicators from 2004 onwards.

### Summary statistics

Because income is recorded in the GP WLS in eight intervals from less than £25,000 to more than £150,000, we recode it as a continuous variable by assigning each doctor the mid value of the income interval they report. The average income in our GP WLS (2004-2005) sample is about £73,311. On average, doctors report to work about 44 hours per week as GP^10^.

We also consider a number of practices’ and doctors’ characteristics from the GMS database (2003-2007). We use FTE of doctors as measure of their effort (i.e. parameter *e*_*zi*_ in the theoretical model). FTE indicates the workload of a doctor on a scale from 0 to 100 where 100 indicates the doctor is employed as full-time worker in the practice and 50 signals that the worker is only half-time. Table 1 reports that on average between 2003 and 2007 doctors work almost 91 percent of their working time. But there is quite a high standard deviation indicating differences between doctors. The FTE in the GP WLS (calculated from the self-reported weekly hours of work) is about 97 percent.

**Table 1:** Description of the variables in the empirical models 2003-2007

Variable	Obs.	Mean	Std. dev.	Min.	Max.
*Outcome variables:*					
Principal	171,212	0.82		0.00	1.00
Mobility (old doctors)	141,930	0.06		0.00	1.00
New doctors	171,212	0.17		0.00	1.00
Exit	171,212	0.06		0.00	1.00
FTE	171,212	90.77	17.93	11.00	100.00
Proportion total QOF	138,303	96.97	5.68	10.00	100.00
points					
*Independent variables:*					
Age	171,212	45.18	9.74	23.00	85.00
Female	171,212	0.42		0.00	1.00
Practice years	171,212	28.92	6.26	0.00	32.99
Total population	171,204	8.77	4.51	0.00	37.61
(/1,000)					
LISI	170,930	11.23	7.41	0.00	90.00
Proportion female	171,212	4.23	1.26	0.00	33.33
65-74					
Proportion female	171,212	4.62	1.73	0.00	16.10
75+					
Proportion male	171,212	3.91	1.17	0.00	33.33
65-74					
Proportion male	171,212	2.89	1.19	0.00	100.00
75+					
Distance to the	171,212	8.76	16.32	0.01	390.33
best practice					
Price shock	35,043	-1.41	3.74	-36.86	0

Note: price shock refers to 2005/06

Contractual status is available in the GMS data and records whether the doctor is partner or salaried. On average in our sample 82 percent of doctors work as partners.

We use the GMC reference code in the GMS data to identify new and old doctors. On average about seven percent of doctors between 2003 and 2007 are newly qualified, about six percent exit the healthcare job market or, if they don’t exit the market, they move practice.

Our measure of quality (i.e. the parameter *Q* in the theoretical model) is practice performance in the QOF as measured by the proportion of total points achieved by the practice. The revenue the practice earns varies linearly between lower and upper thresholds of coverage, called “achievement” *A*∈[0,1]. It indicates the proportion of eligible patients for which a specific measurement of quality has been achieved. No money is received by the practice if achievement is less than or equal to the lower threshold and the maximum revenue is received if the practice is on or above the upper threshold [see Gravelle et al. ([8]); Doran et al. (2011) for further details]. Practices performed quite well as the proportion of total points they achieved in the QOF is almost 97 percent. The low standard deviation indicates small differences between practices.

We select a number of control variables that vary among doctors and practices. On the doctor’s side, we consider gender and age in years. Approximately 42 percent of doctors are female and the average age of doctors is 45 years old (with a standard deviation of 9 years). We show that age indicates doctors’ experience which is an important component of doctors’ efficiency parameter (i.e. parameter *γ*_*z*_).

On the practice’s side, we consider the number of years the practice exists, the size of its population and the proportion of its female and male population aged 65 or over. Practice years is calculated as difference between current and opening date. On average, practices have been in business for about 29 years, but there is a standard deviation of about 6 years. On average, about four percent of the practice population is aged 65 or over. A practice could also have up to a third of its population that is aged 65 or over. We also consider a measure of deprivation based on prescribing in general practice, the LISI (i.e. parameter *b*_*i*_ in the theoretical model). More than 80 percent of prescription by NHS GPs in England are exempt from charges. Most of such exemptions are made on the basis of age and specific diseases. However, 12.1 percent of items are exempt because of low income. LISI includes recipients (and their dependents) of family credit and income support and others who qualify because of low income. It indicates the proportion of total cost in a practice going to patients who are exempt for these reasons. On average, eleven percent of a practice’s cost goes to patients with low income, but there is quite a large variation between practices. Practices in deprived areas may have to incur to 90 percent of their costs to poorer patients.

We use the postcode to obtain the latitude and longitude of each practice. We then calculate the Vincenty (1975) distance to the 10 closest practices. Vincenty’s formula is used in geodesy to calculate the distance between two points on the assumption that the figure of the Earth is an oblate spheroid rather than spherical. We then calculate the distance (in miles) of the most distant practice amongst the closest 10 practices. We select the distance to the best practice (measured by its QOF points) that is the most further away amongst the 10 selected practices. Under the assumption that relocation costs increase with distance, this is an empirical proxy for parameter *t* in the theoretical model. We recognise, however, that attractiveness of a practice is determined by other occupation characteristics (i.e. wages) offered to the doctor that we cannot observe. On average, the best practice is at about nine miles distance, but there is a standard deviation of about 16 miles.

Finally, we select four indicators for the management of cardiovascular diseases (CVD), the only ones for which there was an increase in the threshold levels and/or a decrease in the number of points from 2005 to 2006. This change corresponds to a price decrease as practices were paid less in 2006 if they performed the same as in 2005. These indicators measure whether practices successfully manage CVD by keeping blood pressure and cholesterol under the recommended levels. We aggregate them into a CVD control indicator. For an average practice, these indicators were worth about £5,395 in 2006. We then define the exogenous price shock to be the difference between what practices would receive if they continued to provide the same quality in 2006 as they did in 2005 but with the new 2006 thresholds and what they actually received in 2005. The size of the negative price shock can be as high as 37% and on average practices could lose up to 1% in 2006 if they performed in 2006 the same as they did in 2005.

## Results and discussion

Before we discuss our results we explain how we measure efficiency. In Table 2 we report estimates of Eq. 12, the pooled regression models of practice quality measured as the proportion of total QOF points. We indirectly show that age is a proxy for doctor’s efficiency with three separate models. First, in model I, estimated on the GMS database, we find that in single-handed practices an increase of doctor’s age is associated with a better performance of the practice. But this association is non-linear as it is characterised by an inverted-U shape with a decline after the age of 42. The same non-linear relation between age and quality of the practice can be found in Model II using the full sample of practices. However, one limitation is that age could capture the effect of omitted factors such as income. In Model III, estimated on the GP WLS sample, we show that even after controlling for doctors’ income, age statistically significantly affects practices’ quality. As doctors gain more experience they improve practices performance up to the age of 41 after which their efficiency starts declining.

**Table 2:** Pooled linear models of determinants of practices’ quality (2004–2005)

	GMS:		GP worklife survey:
	Model I	Model II	Model III
Age	0.84***	0.24***	0.33**
	(0.23)	(0.03)	(0.14)
Age squared	-0.01***	-0.003***	-0.004**
	(0.002)	(0.0003)	(0.002)
Female	0.70	0.09*	0.10
	(0.52)	(0.05)	(0.20)
FTE	0.04	-0.01***	-0.01***
	(0.03)	(0.001)	(0.005)
Income	-	-	0.000
			(0.000)
Distance to the best	0.07	0.01**	0.01***
practice	(0.06)	(0.002)	(0.003)
Total population	0.71***	0.12***	0.10***
	(0.25)	(0.01)	(0.03)
LISI	-0.15***	-0.10***	-0.09***
	(0.04)	(0.01)	(0.03)
Proportion female 65-74	0.75**	0.66***	0.39*
	(0.37)	(0.11)	(0.23)
Proportion female 75+	0.06	0.14**	0.27*
	(0.24)	(0.07)	(0.15)
Proportion male 65-74	-0.20	-0.53***	-0.22
	(0.38)	(0.12)	(0.28)
Proportion male 75+	-1.06**	-0.33***	-0.52
	(0.44)	(0.12)	(0.34)
Practice years	-0.04	0.03***	0.03
	(0.02)	(0.01)	(0.02)
Constant	74.19***	92.78***	90.35***
	(6.07)	(0.74)	(3.21)
N. observations	1652	31,551	1971
N. practices	1652	8192	1659

Note: Model I on single practices, Models II-III on all practices

^∗∗∗^ *p*<0.01; ^∗∗^ *p*<0.05; ^∗^ *p*<0.10

We report results of the three testable implications in four tables. The first and second testable implication around doctor efficiency, partnership and mobility are reported in Table 3. Results on the third testable implications are reported in Tables 4, 5 and 6.

**Table 3:** Linear probability models of partnership and mobility (2003–2007)

	Partnership:		Mobility:
	Model I	Model II	Model I
Age	0.07***	0.05***	-0.06***
	(0.001)	(0.001)	(0.01)
Age squared	-0.001***	-0.0004***	0.001***
	(0.00001)	(0.00001)	(0.00005)
Female	-0.06***	-0.12***	0.009***
	(0.003)	(0.004)	(0.002)
Prop. total QOF points	-	-	-0.0004***
			(0.0001)
FTE	0.003***	0.001***	0.001***
	(0.0001)	(0.00004)	(0.0001)
Distance to the best practice	0.0001	-0.00006	-0.00004
	(0.0001)	(0.00003)	(0.00004)
Total population	0.009	0.002***	-0.002***
	(0.001)	(0.0004)	(0.0002)
LISI	-0.0003	-0.001***	0.001***
	(0.0002)	(0.0002)	(0.0002)
Proportion female 65-74	0.01***	0.004**	-0.001
	(0.003)	(0.002)	(0.002)
Proportion female 75+	0.004	0.002**	-0.003***
	(0.003)	(0.002)	(0.001)
Proportion male 65-74	-0.001**	-0.0004	0.003
	(0.003)	(0.002)	(0.002)
Proportion male 75+	-0.01*	-0.0001	0.003*
	(0.003)	(0.001)	(0.001)
Practice years	0.002***	0.002***	-0.001***
	(0.0003)	(0.0003)	(0.0002)
Practice size	-0.02***	-0.01***	-0.002***
	(0.001)	(0.0004)	(0.001)
2004	-0.04***	-0.01***	0.02***
	(0.002)	(0.001)	(0.003)
2005	-0.06***	-0.01***	0.02***
	(0.002)	(0.001)	(0.002)
2006	-0.12***	-0.06***	0.03***
	(0.003)	(0.002)	(0.002)
2007	-0.15	-0.07***	-
	(0.003)	(0.002)	
Inverse Mills’ ratio	-	-	-0.04
			(0.05)
Constant	-1.18***	-0.55***	1.60***
	(0.03)	(0.02)	(0.02)
N. observations	141,529	141,529	141,529
N. practices	8507	8507	8507

Note: Model I is a pooled linear regression and Model II is a random intercept multilevel model

Model I of Mobility is second stage of Heckman model

^∗∗∗^ *p*<0.01; ^∗∗^ *p*<0.05; ^∗^ *p*<0.10

**Table 4:** Linear models of doctors’ effort, practice quality and access of new doctors (2004–2007)

	Model I:	Model II:	Model III:
	FTE	Proportion of total	Access of
		QOF points	new doctors
Age	0.36***	0.17***	-0.01***
	(0.06)	(0.02)	(0.0002)
Age squared	-0.01***	-0.002***	-
	(0.001)	(0.0002)	
Female	-0.12***	0.16***	-0.02***
	(0.002)	(0.05)	(0.003)
Being a partner	-0.07***	-	-
	(0.01)		
Age* Being a	0.004***	-	-
partner	(0.0002)		
Distance to the	-0.02***	0.001	-0.00004
best practice	(0.002)	(0.001)	(0.0001)
Total population	0.50***	0.01	-0.01***
	(0.02)	(0.01)	(0.001)
LISI	0.04***	-0.11***	0.001**
	(0.01)	(0.003)	(0.0002)
Proportion female	0.45***	0.52***	-0.001**
65-74	(0.14)	(0.04)	(0.003)
Proportion female	-0.34***	0.05*	-0.001
75+	(0.08)	(0.02)	(0.002)
Proportion male	0.10	-0.48***	0.004
65-74	(0.13)	(0.04)	(0.003)
Proportion male	-0.05	-0.07**	0.001
75+	(0.09)	(0.03)	(0.004)
Practice years	-0.03***	0.04***	-0.0005*
	(0.01)	(0.004)	(0.0003)
Practice size	-0.58***	0.23***	0.01***
	(0.03)	(0.01)	(0.001)
2005	-0.16**	3.40***	-0.04***
	(0.08)	(0.03)	(0.002)
2006	4.39***	3.54***	-0.06***
	(0.09)	(0.03)	(0.002)
2007	4.09***	3.69***	-0.07***
	(0.94)	(0.03)	(0.002)
Constant	83.25***	90.43***	0.87***
	(1.37)	(0.42)	(0.01)
N. observations	126,930	126,626	102,325
N. practices	8504	8472	5033

Note: Models I and II are linear random intercept multilevel models. Partner and female in Model I are on a 0-100 scale. Model III is a linear probability random intercept multilevel model. ^∗∗∗^ *p*<0.01; ^∗∗^ *p*<0.05; ^∗^ *p*<0.10

**Table 5:** First-differenced models with exogenous price shock (2005–2006)

	Model I:	Model II:
	Change in	Change in access
	FTE	of new doctors
Price shock	0.10***	0.001
	(0.03)	(0.0005)
Change in distance to the	-0.05***	0.001
best practice	(0.01)	(0.0001)
Change in total population	0.40***	-0.02***
	(0.09)	(0.002)
Change in LISI	-0.01	-0.002
	(0.03)	(0.001)
Change in proportion female	1.10**	0.06***
65-74	(0.50)	(0.02)
Change in proportion female	-0.43	0.06***
75+	(0.34)	(0.01)
Change in proportion male	-0.03	0.01
65-74	(0.49)	(0.01)
Change in proportion male	-0.66	-0.16
75+	(0.63)	(0.02)
Change in practice size	-0.16	0.03***
	(0.11)	(0.003)
Constant	3.45***	-0.01***
	(0.14)	(0.003)
N. observations	34,954	25,905
N. practices	8119	4756

^∗∗∗^ *p*<0.01; ^∗∗^ *p*<0.05; ^∗^ *p*<0.10

**Table 6:** First-differenced models with exogenous price shock (2005–2006)

	Model I: bottom quintile	Model II: top quintile
	Change in mobility	Change in mobility
Price shock	-0.0003	-0.0003
	(0.001)	(0.003)
Change of distance to	-0.0001	0.0003
the best practice	(0.0003)	(0.0002)
Change in total	-0.03***	0.01
population	(0.01)	(0.01)
Change in LISI	0.004**	-0.003
	(0.002)	(0.003)
Change in proportion	-0.04	-0.09**
female 65-74	(0.03)	(0.04)
Change in proportion	-0.005	-0.04
female 75+	(0.03)	(0.04)
Change in proportion	0.01	0.02
male 65-74	(0.03)	(0.04)
Change in proportion	0.01	0.16***
male 75+	(0.03)	(0.05)
Change in practice	-0.01*	-0.00004
size	(0.01)	(0.004)
Inverse Mills’ ratio	0.45***	0.13
	(0.11)	(0.12)
Constant	-0.08***	-0.04***
	(0.01)	(0.02)
N. observations	5967	5384
N. practices	2282	1151

^∗∗∗^ *p*<0.01; ^∗∗^ *p*<0.05; ^∗^ *p*<0.10

In Table 3 we report two models of the partnership equation that show results of the first testable implication. Model I is a pooled linear regression and Model II is a random intercept multilevel model where doctors are nested in practices. Both models show that more efficient doctors, where efficiency is proxied by age, are more likely to become partners up to the age of 35 after which the probability of becoming a partner declines. Model II indicates that with a one year of additional experience there is an increase in the probability of becoming a partner by five percentage points when ability is accounted for, compared to seven percentage points when ability was unaccounted for in Model I. Effort is a statistical significant factor in explaining the probability to become a partner. We show that the introduction of the QOF in 2004 decreased the probability of becoming a partner by one percentage point.

There is no evidence of sample selection in the mobility equation^11^. Mobility decreases with age as presumably the costs of moving become higher. An additional year decreases the probability of moving by six percentage points. A doctor working in a practice that performs better in terms of QOF points is less likely to move. A ten percentage point increase in the proportion of total QOF points decreases the probability of moving practices by 0.4 percentage point. But we observe a raise of mobility over time, in 2004 with the introduction of the QOF there is an increase in the probability of moving by two percentage points.

Models I-III in Table 4 report the estimates for the third testable implication on the medical effort, the quality of the practice and access of new doctors in the market. We find evidence of a decrease in medical effort (variable *e*_*zi*_ in the model) associated with the increase in the QOF price introduced in 2005. In Model II we find however an increase in the proportion of total QOF points achieved by practices. This result may be explained by the possible effects that forms of *intrinsic motivation* play in the market. Indeed theoretical contributions (Bénabou and Tirole [1]) and industry practioners^12^ have argued that doctors’ effort may not simply be the result of financial decisions, but also the product of social/reputational considerations. If reputation and pro-social behaviour is important to doctors, the introduction of a P4P scheme may induce doctors to reduce their effort in those tasks that receive a financial incentive to avoid being recognised as greedy. Practice management instead may be less affected by reputational/pro-social considerations, and may react positively to the introduction of a P4P scheme. In model I there is also evidence that more efficient doctors work more, but this association declines as they get older. Effort provision varies by contractual arrangement as more efficient partners put more effort. The more distant the nearest best QOF practice is, the less effort doctors put, indicating either less competition or lower demand for services.

Finally, in Model III we report estimates of the linear probability model of access to the market of new doctors. All covariates are contemporaneous because we don’t observe the characteristics of doctors prior to their entrance in the healthcare job market. In this specification we compare 2005 with 2004 as we want to test the effect of a price increase in the QOF on the likelihood that doctors enter the market. We show there is no evidence that an increase in the QOF price in 2005 has increased doctors’ access to the healthcare job market. Actually, compared to 2004 in 2005 the probability that a new doctor enters the market *decreases* by four percentage points.

The results in Tables 5 and 6 report the effect of the price shock in 2005. We find results symmetric to the previous ones with an increase of doctors effort and access to the market of new doctors, although the latter is not statistically significant. There is statistically significant effect on mobility for either top or bottom practices based on their performance in 2004. We replicate this analysis using the exogenous price shock on all CVD indicators and find similar results which are available on request.

In Table 7 in the Appendix we show that our results on partnership, mobility and access to the healthcare job market are unaffected by the linear specification because the coefficients of the non-linear models are qualitatively similar.

## Conclusions

There is a lack of research on the sorting and retention effects that P4P may produce. In this paper we focus on the only P4P scheme for General Practitioners in England, the Quality and Outcomes framework (QOF).

We have developed a simple two-practice theoretical model where we account for doctors’ altruism, different contractual arrangements and multidimensional quality. The model predicts more efficient doctors should be more likely to be offered a partnership. The QOF may induce less turn-over and generate more mobility among more efficient doctors.

We have then tested the empirical implications of this model using a unique linkage between doctors Census data from 2003 to 2007, practices’ characteristics and quality as measured by the QOF in England. After controlling for doctors’ unobserved heterogeneity in multilevel models where doctors are nested in practices, we have found that the QOF is associated with a two percentage point increase in mobility. We have also found that the introduction of the QOF is associated with a decrease in the probability of becoming a partner by one percentage point, but more efficient doctors are more likely to become partners. More efficient doctors improve the quality of the practice and if they are also partners they put more effort.

Despite the relative richness of the data, our study has some limitations. Firstly, as the QOF applied to all practices in England we identify its effect off time dummies. However, the National Audit Office Report [11] does not highlight any policy other than the QOF that was introduced in England between 2004 and 2005. We have also checked the robustness of our results using the 2006 negative price shock which is exogenous to the individual doctor’s behaviour and we found similar results. Secondly, we cannot measure wages at the same time as mobility. We had to resort to an indirect method arguing that age can proxy for experience, a component of efficiency. We have shown this holds in single handed practices where practice quality is doctor’s quality. However, this relies on the assumption that the only factor that affects practices’ quality is doctors’ effort and not the effort of other staff.

## Endnotes

^1^ See for example Glazer [7]. In the rest of the paper will use the terms devotion, vocation, motivation and altruism interchangebly.

^2^ The assumption that the cost of effort is described by a quadratic function is commonly adopted in the literature (see for example Brekke and Sørgard [3] and Zweifel et al. [18]). The convexity of the cost function captures an opportunity cost concept, i.e. the idea that an additional unit of time/effort spent at work increases the value of leisure.

^3^ The assumption that health care providers face a convex function is standard. Convexity captures that idea that practices face a capacity constraintthere are physical limitations in physical resources. See for example Brekke et al. [2].

^4^ If the doctors had an outside option ensuring a monetary reward greater than zero, then practices would have to consider the satisfaction of a participation constraint and, in case of a binding constraint, the fact that equilibrium profits would depend on the monetary remuneration of the outside option. Qualitatively the results of the model remain unchanged.

^5^ The assumption that the partners of a practice equally share profits is clearly a simplification. Nonetheless, given the lack of information in the data available to us, it is an innocent assumption.

^6^ The opposite case where type *v* is located next to practice 2 is completely symmetric to case (iii).

^7^ A very recent debate in the news seems to point out that *m* may have been reduced in the last few years. http://www.bbc.co.uk/news/uk-politics-24362902.

^8^ Multilevel models are used because doctors are nested in practices.

^9^ We test the exclusion restriction assumption by including the number of years to statutory retirement directly in the mobility equation. We find that it does not statistically significantly affect mobility.

^10^ These statistics are not reported in Table 1 as they are not part of our main analysis.

^11^ We do not report the results of the year-by-year first stage probit selection model, but these can be made available on request.

^12^ http://www.telegraph.co.uk/news/health/news/11177632/Anger-over-cash-for-diagnoses-dementia-plan.html

## Appendix A: Additional model specifications

**Table 7:** Probit models of partnership, mobility and access of new doctors)

	Model I	Model II	Model III
	Partnership:	Mobility:	Access of
			new doctors:
Age	1.03***	-0.33***	-0.22***
	(0.02)	(0.01)	(0.01)
Age squared	-0.01***	0.003***	-
	(0.0002)	(0.00001)	
Female	-1.48***	0.03	-0.07
	(0.04)	(0.02)	(0.04)
Prop. total QOF points	-	-0.01***	-0.001
		(0.002)	(0.001)
FTE	0.02***	0.003***	-
	(0.001)	(0.001)	
Distance to the best practice	0.002*	-0.001	-0.001
	(0.001)	(0.001)	(0.001)
Total population	0.13***	0.02***	-
	(0.01)	(0.005)	
LISI	0.004	0.002	0.01**
	(0.003)	(0.001)	(0.003)
Proportion female 65-74	0.16***	-0.02	-0.13***
	(0.05)	(0.02)	(0.05)
Proportion female 75+	0.05*	-0.04***	-0.01
	(0.03)	(0.01)	(0.03)
Proportion male 65-74	-0.08*	0.05**	0.09*
	(0.05)	(0.02)	(0.05)
Proportion male 75+	-0.06	0.02	0.03
	(0.05)	(0.01)	(0.06)
Practice years	0.02***	-0.008***	-0.01**
	(0.003)	(0.001)	(0.004)
Practice size	-0.23***	-0.06***	0.17***
	(0.01)	(0.01)	(0.009)
2004	-1.96***	-0.01***	-
	(0.18)	(0.001)	
2005	-2.02***	-0.01***	-0.88***
	(0.18)	(0.001)	(0.03)
2006	-3.00***	-0.06***	-1.65***
	(0.19)	(0.002)	(0.05)
2007	-3.35	-0.07***	-2.68***
	(0.19)	(0.002)	(0.07)
Inverse Mills’ ratio	-	5.25***	
		(0.62)	
Constant	-20.64***	6.96***	7.49***
	(0.03)	(0.25)	(0.21)
N. observations	141,529	126,626	102,325
N. practices	8507	8507	5033

^∗∗∗^ *p*<0.01; ^∗∗^ *p*<0.05; ^∗^ *p*<0.10
